# Oral health care professionals recommending and administering the HPV vaccine: Understanding the strengths and assessing the barriers

**DOI:** 10.1371/journal.pone.0248047

**Published:** 2021-03-04

**Authors:** Denise Guadiana, Nolan M. Kavanagh, Cristiane H. Squarize

**Affiliations:** 1 Department of Periodontics and Oral Medicine, University of Michigan School of Dentistry, Ann Arbor, Michigan, United States of America; 2 Perelman School of Medicine, University of Pennsylvania, Philadelphia, Pennsylvania, United States of America; 3 Laboratory of Epithelial Biology, Department of Periodontics and Oral Medicine, University of Michigan School of Dentistry, Ann Arbor, Michigan, United States of America; 4 University of Michigan Rogel Cancer Center, University of Michigan Medical School, Ann Arbor, Michigan, United States of America; Universita Politecnica delle Marche, ITALY

## Abstract

**Introduction:**

Head and neck cancer is a deadly cancer that ranks among the six most common cancers worldwide. The HPV vaccine has been used to prevent head and neck cancer of the oropharynx, and changes in health policies and state law are impacting the role of dental professionals in HPV vaccination. However, relatively little is known about dental professionals’ attitudes regarding the vaccine.

**Objectives:**

Our study assesses dental professionals’ willingness to administer the HPV vaccine, their confidence discussing HPV with patients, beliefs about the vaccine’s efficacy, perceived barriers to administering it, and sites of referral.

**Methods:**

We surveyed 623 dental professionals, including dentists, hygienists, dental students, and hygiene students across Michigan. Attitudes toward the vaccine and predictive characteristics were evaluated by logistic regression, ANOVAs, and t-tests.

**Results:**

The majority of the respondents (51% of dentists, 63% of hygienists, 82% of dental students, and 71% of hygiene students) were willing to administer the HPV vaccine if allowed by law. The role of dental and dental hygiene students would be one of advocacy, educating and recommending the vaccine, and the dental students administering it once licensed. Dental professionals were variably confident discussing HPV with patients and generally believed it enhanced patients’ health. Stronger confidence and beliefs were associated with greater willingness to administer the vaccine. Barriers among professionals opposing the HPV vaccine included lack of knowledge on the subject, liability concerns, and personal beliefs.

**Conclusion:**

Dental professionals can become leaders in preventing HPV-related cancers. Training and continuing education courses could enhance their confidence and willingness to recommend and administer the HPV vaccine.

**Policy implications:**

Legislation that permits dental professionals to administer the vaccine could increase the vaccine’s accessibility to patients, improve vaccination rates, and population health.

## Introduction

The human papillomavirus (HPV) is associated with the development of head and neck cancer, particularly in the oropharynx (tonsils, base of the tongue, and throat) and floor of the mouth [1]. An estimated 80% of all sexually active people are infected with some strain of HPV [1]. Two high-risk strains, HPV-16 and HPV-18, are commonly found in premalignant lesions and tumors. Indeed, high-risk HPV contributes to 9–42% of the floor of the mouth cancers and 70–90% of oropharyngeal cancers (OPC) [2–5]. OPC is now the most common HPV-associated cancer in the United States, surpassing cervical cancer [6]. This upsurge has made urgent a discussion about an interprofessional approach that includes oral health care professionals in cancer prevention. Both the American Dental Association (ADA) and the American Academy of Pediatric Dentistry (AAPD) urge oral health care providers to participate in the prevention of oropharyngeal cancer through education and advocacy for HPV vaccination [7, 8].

HPV-related cancer can be prevented through vaccination. Since 2006, multiple HPV vaccines (2vHPV-Cervarix, 4vHPV-Gardasil, and 9vHPV-Gardasil 9) have been licensed in the U.S. Currently, the nine-valent vaccine (9vHPV-Gardasil 9) is the only one available in the U.S. [9]. Notably, the HPV vaccine is effective against the high-risk subtypes of the virus and reduces oral HPV infection rates [10–12]. Indeed, studies showed a 93% reduction in oral HPV infections among young adults who received at least one dose of the vaccine [12–14]. However, among all available vaccines, the HPV vaccine has the highest rate of parental declination and hesitancy [15]. Although uptake among adolescents is gradually increasing, its rates remain below optimal levels compared to other vaccines [16]. As of 2017, only about 50% of eligible adolescents have received the complete vaccine series [15].

A health care provider’s recommendation is one of the primary reasons that parents agree to HPV vaccination of their children [17–20]. Approximately 85% of children visit dental providers each year; and most visits are on a biannual basis [21, 22]. The wide reach of dental professionals and the frequency of visits represent an opportunity to recommend the HPV vaccine or even provide the HPV vaccine in the recommended 2-dose schedule. Changes in public health policies and state laws now allow dentists to administer vaccines in 3 U.S. states, Oregon (for all vaccines), Minnesota, and Illinois (for the flu vaccine in adults), as well as abroad (Scotland) [23–26]. This emerging role for oral health care providers has prompted studies evaluating their knowledge and confidence in discussing HPV prevention and its link to OPC with patients [27–39]. Nonetheless, the dental professionals’ active role and attitudes toward the vaccine is poorly understood.

The present study aimed to determine the attitudes of dental professionals towards recommending and administering the HPV vaccine. The primary aim was to assess and compare the willingness of dental professionals in various roles to administer the HPV vaccine. These roles included registered dentists and hygienists as well as dental and hygiene students. Oral health care providers participate in secondary prevention methods (e.g. oral cancer screenings and patient education); and aligned with ADA and AAPD statements, their role in cancer prevention is being expanded to include the vaccine, whether by administering it (as for dentists) or recommending it (hygienists) [30, 33]. We also evaluated their confidence discussing HPV with patients and beliefs, barriers, and referral sites regarding the vaccine. The results shed light on policy and educational improvements that could empower dental professionals to prevent the spread of HPV and the rise of HPV-related cancers.

## Materials and methods

### Study population and questionnaire

We conducted a cross-sectional study using a 17-item survey of attitudes toward administering and recommending the HPV vaccine. It was distributed to dental professionals (registered dentists, registered dental hygienists, dental students, and dental hygiene students) in Michigan. The survey consisted primarily of Likert-scale questions (for attitudes). Three 5-point Likert scales were used to ask respondents about their confidence, beliefs, and attitudes regarding the HPV vaccine; the first scale ranged from very unlikely to very likely, the second from completely uncertain to completely confident, and the third from strongly disagree to strongly agree. For barriers to administering the vaccine, respondents could choose between options we provided or write their own. The development of the questionnaire was based in surveys obtained from the publications [27, 31, 34]. Survey questions were developed and reviewed with a specialist from the University of Michigan Survey Research Center, who evaluated the survey for face and content validity. Online survey was distributed online using Qualtrics (Qualtrics XM). Two dentists and two dental hygienists piloted the survey, whose feedback was used to revise and finalize the survey. The survey questions are available in the S1 Table.

The survey was distributed to dental professionals in Michigan as follows: For dentists, a random sample of 650 registered dentists was mailed paper surveys, using addresses available in the statewide Department of Licensing and Regulatory Affairs (LARA) directory. 140 dentists responded in total, for a response rate of 22%. For hygienists, participants were recruited at the Dental Hygiene Education Annual Conference (Lansing, MI); of its approximately 100 attendees, we received 91 respondents, or 91%. For dental and hygiene students, electronic surveys were developed in Qualtrics and emailed to directors of the two dental schools and all 12 hygiene programs in the state. Directors were asked to forward the survey to their students. Moreover, hard copies were delivered to both dental schools and available to hygiene students in attendance of the Dental Hygiene Education Annual Conference. Of the 989 dental students in the state, 242 responded, or 24%, and of the approximately 650 hygiene students, 150 responded, or 23%. Participants were included if 18 years or older and either licensed professionals in Michigan or students actively enrolled in a dental or hygiene education program in the state. Information provided along with the study explained to the participants that the responses were for research purposes. Responses were gathered anonymously. Participants’ consent was obtained with the completion of the survey. The University of Michigan’s Institutional Review Board approved the study (HUM00129698).

### Statistical analysis

All statistical analyses were performed in R (v. 3.6.1, The R Foundation). Categorical variables (e.g., willingness to administer the vaccine) were analyzed by Chi-squared tests or Fisher’s exact tests when expected cells contained fewer than five respondents. In multivariable analyses, binomial logistic regression was used. For continuous variables (e.g. confidence discussing HPV), one-way analyses of variance (ANOVA) were performed with Tukey’s post-hoc tests for multiple comparisons; for subgroup analyses (e.g. confidence x previous class on HPV), Welch’s two-sample t-tests were used. Likert scales were treated as continuous variables with 1–5 ranges. For all tests, a two-sided *p*-value of less than 0.05 was considered significant. Graphics were created using GraphPad Prism 7 (v. 8.0.2, GraphPad Software, San Diego, CA) and Microsoft Excel (v. 16.22, Microsoft, Redmond, WA). The age of one participant included a typo and was deleted by investigators. Participants were included in analyses if they reported both their professional role and their willingness to administer the HPV vaccine; otherwise, missing data were deleted pairwise. Listwise deletion and multiple imputations (with five imputations) produced comparable results (not shown). The pairwise results are presented.

## Results

### Description of the study population

In total, 623 dental professionals in Michigan responded to the survey. There were 140 dentists, 91 hygienists, 242 dental students, and 150 hygiene students. The respondents comprised 407 females (65%) and 216 males (35%). The respondents’ ages ranged from 19 to 72, with an average of 32.6 and median of 26, reflecting the many students in the survey. Between 30% and 44% of each professional role had taken a previous class on HPV infection (**Table 1**).

**Table 1:** Characteristics of respondents. Dental professionals across Michigan were surveyed about their willingness to administer the HPV vaccine and related attitudes in 2017–2018.

	Dentists (n = 140)	Hygienists (n = 91)	Dental students (n = 242)	Hygiene students (n = 150)
**Female**	51 (36%)	91 (100%)	123 (51%)	142 (95%)
**Male**	89 (64%)	0 (0%)	119 (49%)	8 (5%)
**Age** (mean ± SD)	46.4 ± 14.0* (n = 136)	45.9 ± 13.4 (n = 89)	25.1 ± 3.1 (n = 239)	24.2 ± 4.7 (n = 150)
**Year in school**				
First			127 (52%)	36 (24%)
Second			26 (11%)	56 (37%)
Third			72 (30%)	40 (27%)
Fourth			17 (7%)	18 (12%)
**Years in practice**				
0–5	10 (7%)	15 (16%)		
6–10	18 (13%)	9 (10%)		
11–16	8 (6%)	7 (8%)		
17–20	8 (6%)	7 (8%)		
21–24	11 (8%)	9 (10%)		
25 or more	57 (40%)	39 (43%)		
**Graduate students**	28 (21%)	5 (5%)		
**Approximate percentage adolescents in practice**				
0%	4 (4%)	2 2%)		
10%	46 (41%)	26 (30%)		
25%	35 (31%)	31 (36%)		
50%	9 (8%)	6 (7%)		
75%	8 (7%)	5 (6%)		
100%	2 (2%)	1 (1%)		
**Not practicing**	8 (7%)	15 (17%)		
**Previous class on HPV**				
No	95 (68%)	62 (70%)	135 (56%)	95 (64%)
Yes	45 (32%)	27 (30%)	106 (44%)	53 (36%)

*The age of one participant was deleted due to a typo.

The rates vary by professional role, as shown by a Chi-squared test (**P = 0.01).

### Willingness to administer, confidence, and beliefs about the HPV vaccine

**Fig 1:**
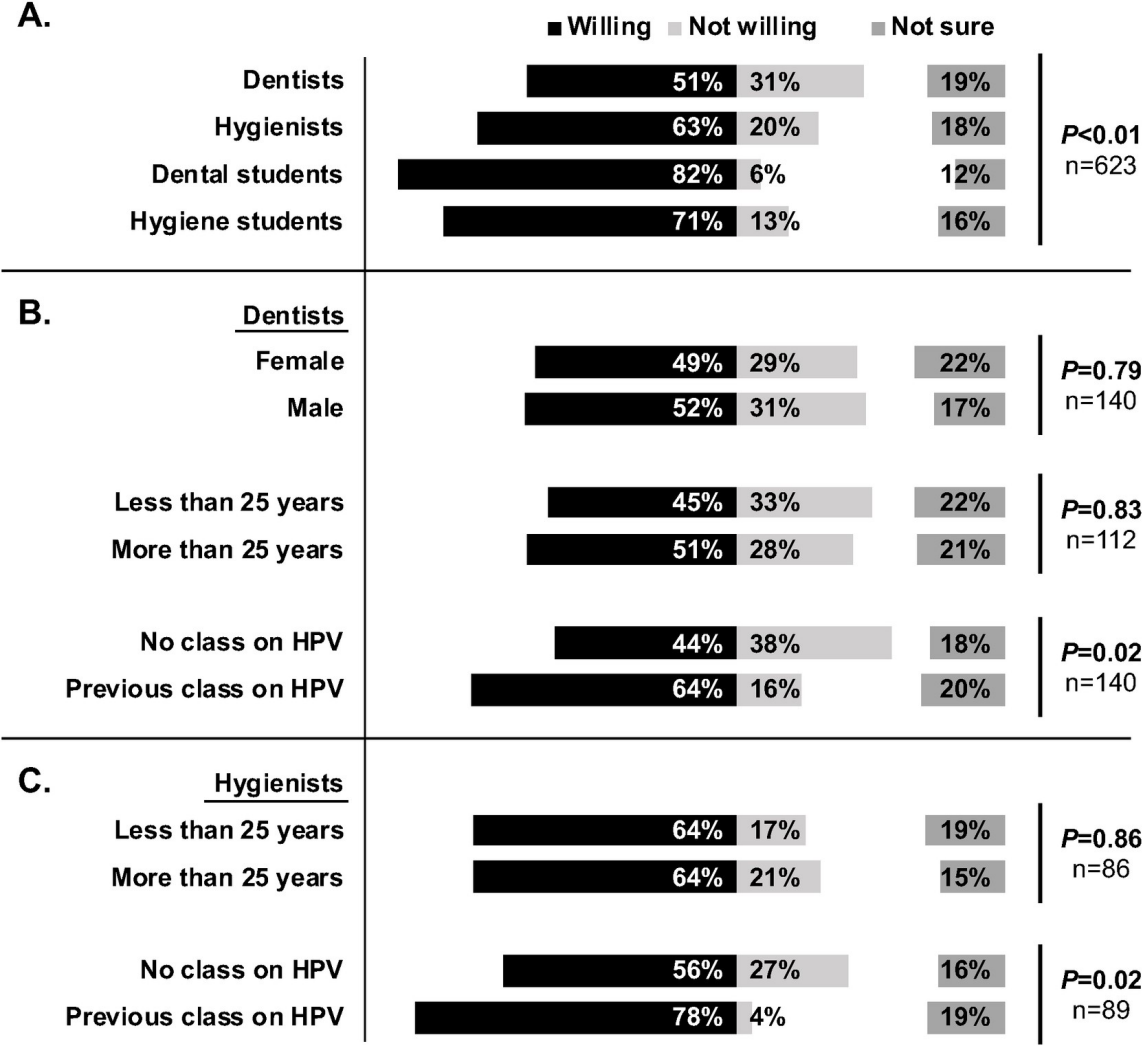
Most dental professionals are willing to administer the HPV vaccine. **(A)** Willingness of dental professionals across Michigan to administer the HPV vaccine if allowed by law (n = 623). Rates vary by professional role, as shown by a Chi-squared test of independence (**P<0.01). **(B)** Willingness among dentists, compared by gender, years in the profession (±25), and having taken a class on HPV infection (n = 140 registered dentists’ responses; graduate students excluded). **(C)** Similarly, as in **(B)**, for registered dental hygienists. All values are percentages. N-values for each analysis are shown. *P*-values for Chi-squared tests or Fisher’s tests, as appropriate, are given. Note that participation in previous educational classes about HPV correlates with the oral health care professional’s willingness to administer the vaccine.

A majority of respondents reported that they would be willing to administer the HPV vaccine if allowed by law: 51% of dentists, 63% of hygienists, 82% of dental students, and 71% of hygiene students (**Fig 1A**). Students were significantly more willing to administer the vaccine than their practicing colleagues (*P*<0.01). Between 12% and 19% of respondents, depending on professional role, were unsure, and only 6% to 31% of the respondents were unwilling. Among practicing dentists and hygienists, the willingness to administer the vaccine was not associated with their gender (*P* = 0.79) or with their number of years in practice (*P* = 0.83). (**Fig 1B and 1C**). However, dentists and hygienists who had previously taken a class on HPV infection and OPC were 20 and 22 percentage points more willing to administer the vaccine, respectively. In other words, 64% of dentists who had taken a previous class on HPV vs. 44% of dentists without a previous class were willing (*P* = 0.02), and 78% of hygienist with a previous class vs. 56% without a previous class were willing (*P* = 0.02) (**Fig 1B and 1C)**. These results suggest that knowledge about HPV and the vaccine impact the professionals’ decision-making.

**Fig 2:**
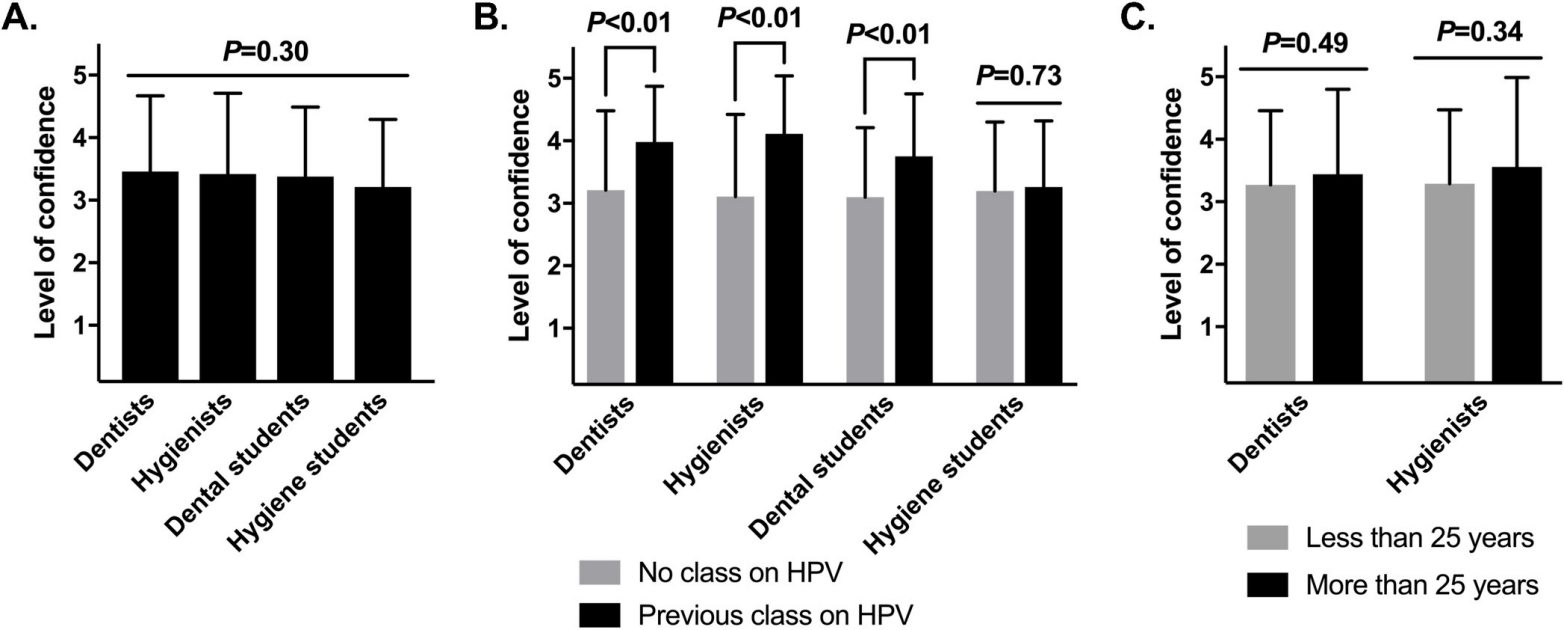
Dental professionals are variably confident in discussing HPV infection with their patients. **(A)** Confidence of dental professionals to discuss HPV infection with their patients. For this analysis, a Likert scale of confidence, ranging from completely uncertain to completely confident, was converted to a 1–5 scale. Means ± standard deviations are shown and were compared by one-way ANOVA (n = 619 respondents; *P* = 0.30). **(B)** Confidence among each professional role, analyzed by having taken a previous class on HPV infection or not (n = 140, 89, 241, and 148, respectively). **(C)** Confidence of licensed professionals, analyzed by years in practice (n = 112 and 84, respectively; graduate students excluded). For **(B)** and **(C)**, means ± standard deviations are shown and were compared within each professional role by Welch’s two-sample t-tests, pairwise. Note that participation in previous educational classes is significantly associated with higher confidence of most oral health care professionals to talk about HPV with patients.

Overall, dental professionals were somewhat confident about talking to patients and parents about HPV infection and prevention (**Fig 2A**). Dentists expressed an average confidence level of 3.5 (SD = 1.2, median = 4, range of 1–5) on a 1–5 Likert scale, or slightly more confident than neutral; for hygienists, 3.4 (SD = 1.3, median = 4, range of 1–5); for dental students, 3.4. (SD = 1.1, median = 4, range of 1–5); and for hygiene students, 3.2 (SD = 1.1, median = 3, range of 1–5). Confidence did not vary significantly by professional role (**Fig 2A**), nor by dentists’ and hygienists’ years in practice (**Fig 2B**). However, it was considerably higher among dentists, hygienists, and dental students that had attended a class on HPV infection (**Fig 2C**). Dentists who had taken a class were 0.8 points more confident than dentists who had not; hygienists, 1.0 point more confident; dental students, 0.6 points more confident, all statistically significant.

**Fig 3:**
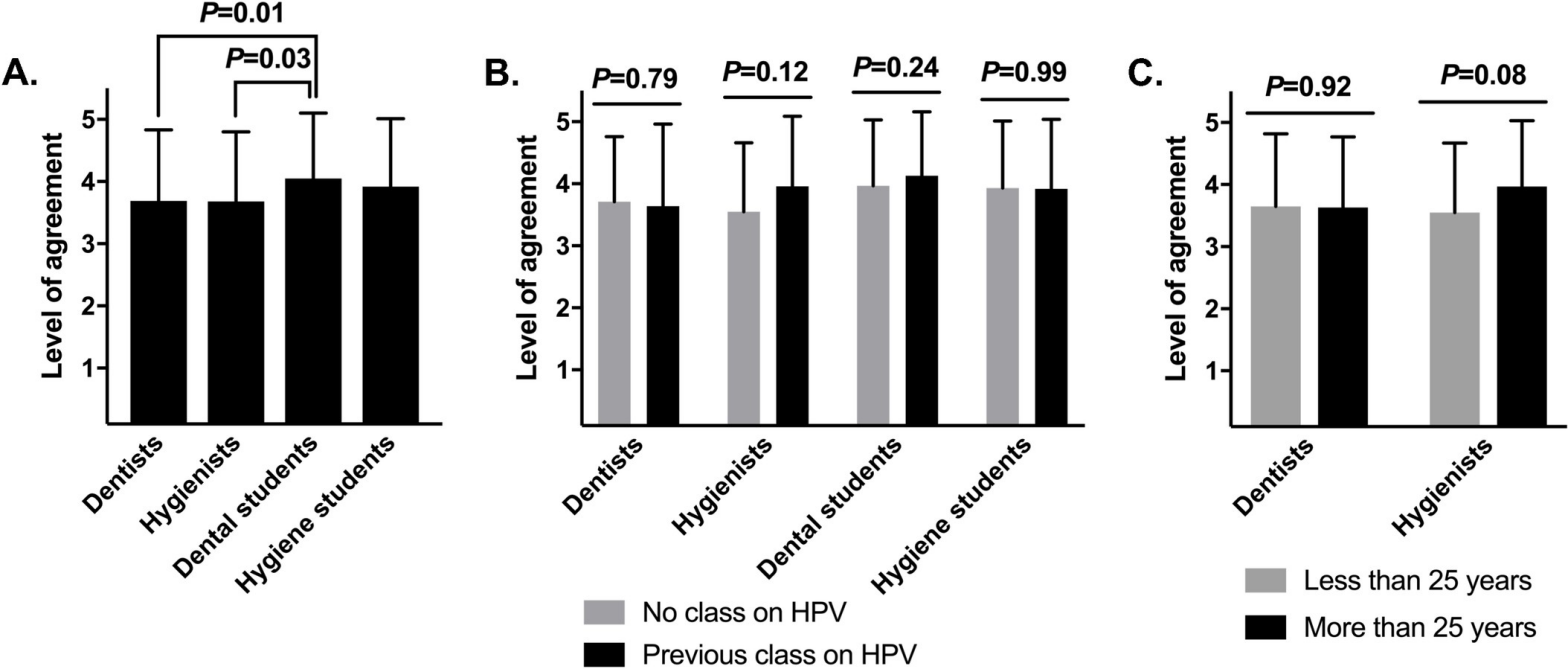
Dental professionals believe that the HPV vaccine enhances the health of patients. **(A)** Agreement that the HPV vaccine improves the health of patients. These analyses used a Likert scale level of agreement, from completely agree to completely disagree, converted to a 1–5 scale. Means ± standard deviations are shown and were compared by one-way ANOVA (n = 623 professionals surveyed; **P<0.01). Significant Tukey’s post-hoc comparisons are shown; non-significant comparisons are excluded. **(B)** Agreement among each professional role, analyzed by having taken a previous class on HPV infection or not (n = 140, 89, 241, and 148, respectively). **(C)** Agreement of dental professionals, analyzed by years in practice (n = 112 and 86, respectively; graduate students excluded). For **(B)** and **(C)**, means ± standard deviations are shown and were compared within each professional role by Welch’s two-sample t-tests, pairwise.

Dental professionals tended to believe that the HPV vaccine can enhance the health of their patients. Dentists expressed an average level of agreement of 3.7 (SD = 1.1, median = 4, range of 1–5) on a 1–5 Likert scale; hygienists, 3.7 (SD = 1.1, median = 4, range of 1–5); dental students, 4.1 (SD = 1.1, median = 4, range of 1–5); and hygiene students, 3.9 (SD = 1.1, median = 4, range of 1–5) (**Fig 3A**). Dental students agreed significantly more than dentists or hygienists (*P*<0.05). Interestingly, these beliefs were not shaped by previous classes on HPV or years in practice (**Fig 3B and 3C**).

In multivariable analyses, binary logistic regressions explored which demographic characteristics, attitudes, or experiences best predicted a professional’s willingness to administer the vaccine (**Table 2**). These analyses excluded the 95 professionals (15% of the total sample) that were unsure whether they would be willing to administer the vaccine. Across the models that included demographics, neither gender nor age significantly predicted willingness. In the fully specified model (model 3), men had 1.46 times the odds of being willing than women (95% CI, 0.74–2.87, not statistically significant), and each additional year of age had an odds ratio of 1.00 (95% CI, 0.98–1.02). These results, in line with the analyses above, suggest that demographic experiences and identities did not noticeably influence professionals’ attitudes toward the HPV vaccine.

**Table 2:** Characteristics that predict Michigan dental professionals’ willingness to administer the HPV vaccine. 528 professionals were surveyed in 2017–2018.

	Model 1 (n = 528)		Model 2 (n = 519)		Model 3 (n = 517)	
	Odds ratio	95% CI	Odds ratio	95% CI	Odds ratio	95% CI
Hygienist^1^	1.92	(1.00–3.68)	**2.39**	**(1.10–5.20)**	**2.72**	**(1.20–6.19)**
Dental student^1^	**8.61**	**(4.44–16.68)**	**9.31**	**(4.16–20.82)**	**8.92**	**(3.85–20.65)**
Hygiene student^1^	**3.41**	**(1.84–6.33)**	**4.06**	**(1.79–9.21)**	**4.49**	**(1.90–10.61)**
Male^2^			1.21	(0.64–2.29)	1.46	(0.74–2.87)
Age			1.00	(0.98–1.02)	1.00	(0.98–1.02)
Previous class					1.33	(0.76–2.33)
Confidence discussing					**1.30**	**(1.04–1.61)**
Belief enhance health					**1.48**	**(1.21–1.81)**
Intercept	**1.65**	**(1.13–2.41)**	1.36	(0.46–4.03)	**0.12**	**(0.03–0.51)**
Nagelkerke R^2^	0.151		0.157		0.233	

^1^Reference group = Dentists.

^2^Reference group = Females. The age of one participant was deleted due to a typo.

These models are binary logistic regressions. “Not sure” answers are excluded. Odds ratios are provided, along with their 95% Wald confidence intervals. Model 1 includes only professional identity; Model 2 adds demographic characteristics, and Model 3 adds confidence, beliefs, and previous classes on HPV. Significant intervals (i.e. those that exclude 1.00) are bolded.

By contrast, stronger confidence discussing HPV with patients and stronger beliefs that the vaccine enhances patients’ health both predicted greater willingness to administer the vaccine. This finding was important to note, although not surprising. In the fully specified model, increasing confidence discussing HPV by one point (along the 5-point scale) increased the odds of willingness by a factor of 1.30 (95% CI, 1.04–1.61), and a one-point increase in the belief that the vaccine enhances patients’ health increased the odds of willingness by a factor of 1.48 (95% CI, 1.21–1.81). Interestingly, confidence and belief that the vaccine enhances health largely attenuated the effect of having taken a previous class on HPV, rendering it a non-significant predictor. That is, a previous class increased the odds of willingness by a factor of 1.33 (95% CI, 0.76–2.33, not statistically significant). This finding suggests that various training models could be effective at boosting confidence to recommend the HPV vaccine and the wiliness to vaccinate.

### Barriers among dental professionals unwilling to administer the HPV vaccine

**Fig 4:**
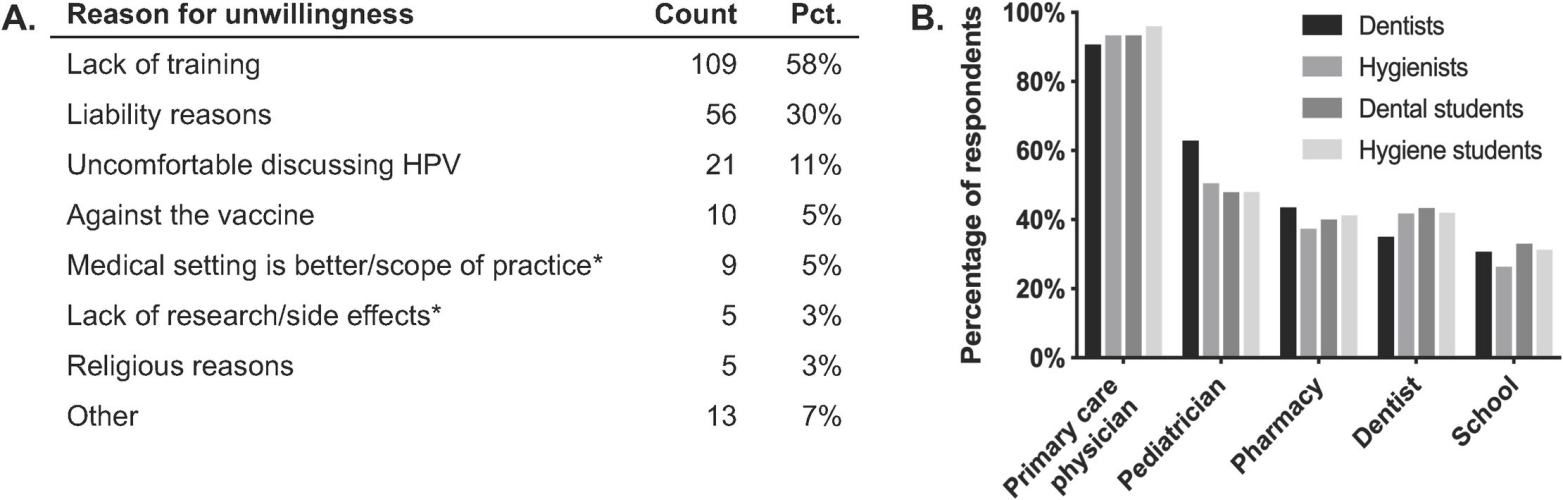
Barriers to administering the HPV vaccine for dental professionals and sites of referral. Ninety-four professionals were unwilling to administer the HPV vaccine and 95 were uncertain. **(A)** Among them, counts and percentages for reasons that these professionals were unwilling or uncertain. *These reasons were written in by participants in the "Other" field. **(B)** Percentages of all survey respondents who were willing to refer their patients to the indicated HPV vaccination site. Percentages were determined using the total number of respondents (n = 623).

Overall, 94 dental professionals (15%) were unwilling to administer the vaccine, and 95 (15%) were unsure. They were asked to indicate all their reasons for being unwilling or unsure. One-hundred and nine respondents (58%) indicated that they would like additional training on the vaccine; this reason was most common (**Fig 4A**). Dental professionals also disclosed concerns about liability (56, or 30%), discomfort discussing HPV infections (21, or 11%), and personal opposition to the vaccine (10, or 5%). These barriers underscore the importance of confidence and knowledge in the previous analyses. Also, some professionals (9, or 5%) stated that the vaccine was outside their scope of practice and/or that medical settings were more appropriate for it.

### Referrals and other attitudes toward the vaccine

Despite the hesitancy of some professionals to administer the vaccine, however, nearly all respondents were happy to refer patients elsewhere to receive the vaccine, even if they themselves were unwilling to administer it. 90–96% of respondents, depending on role, agreed that they would refer patients to primary care physicians (**Fig 4B**), and many would refer to pediatricians (48–63%), pharmacies (37–44%), other dentists (35–43%), or patients’ schools (26–33%). Some respondents noted additional referrals sites, including local health departments and outreach events.

**Fig 5:**
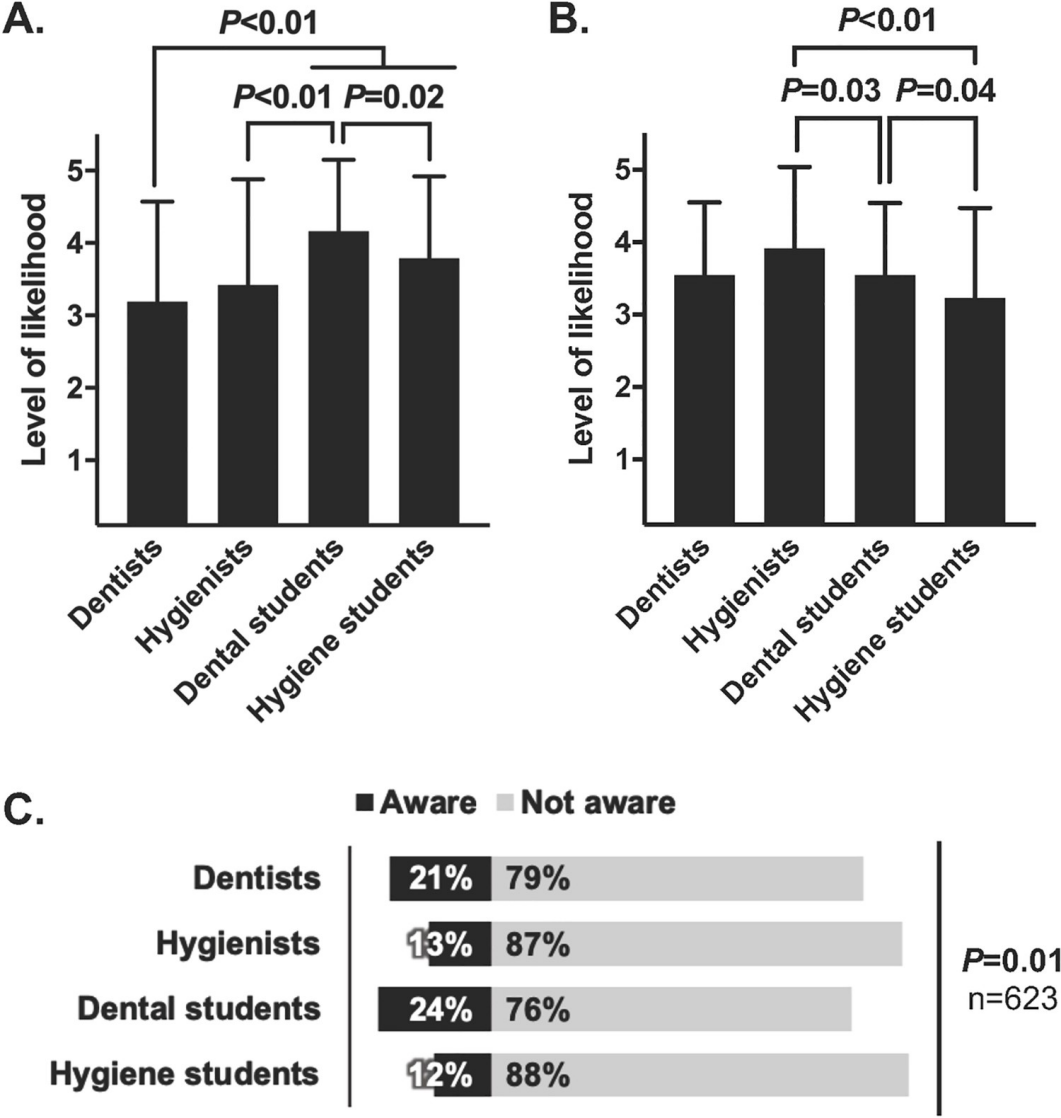
Additional attitudes of dental professionals about the HPV vaccine. **(A)** Likelihood of considering administration of the HPV vaccine if given adequate insurance reimbursement (n = 620). Note that dental professionals generally agree that they’re likelier to administer the vaccine if reimbursed by insurance. **(B)** Likelihood of enrolling in a future class on HPV infection (n = 619). For **(A)** and **(B)**, respondents indicated likelihood along a Likert scale ranging from very unlikely to very likely, which was converted to a 1–5 scale. Means ± standard deviations are shown and were compared by one-way ANOVA (**P<0.01 for **A** and **B**). Significant Tukey’s post-hoc comparisons are shown; non-significant comparisons are excluded. **(C)** Awareness of dental professionals that some states allow dentists to administer vaccines. All values are percentages.

Moreover, it appeared that some of the professionals’ barriers to administering the vaccine may not be immutable. When were asked if they would consider administering the vaccine if given adequate insurance reimbursement, a majority of professionals “somewhat” or “strongly” agreed. Dental students were especially likely to agree (**Fig 5A**). Also, more professionals than not reported being likely to attend a future continuing education course on HPV (**Fig 5B**). This information is notable since one of the major reasons for unwillingness to administer the vaccine was lack of training.

Lastly, respondents were asked of their awareness of an existing care delivery model in which dentists administer vaccines. In some states, such as Oregon, Illinois, and Minnesota, dentists can administer the flu vaccine or other vaccines. However, only 12% to 24% of respondents were aware of this model, depending on their professional role (**Fig 5C**).

## Discussion

As state policies continue to expand the role of dental professionals in HPV vaccination, a clearer understanding of their attitudes and barriers to administering the vaccine is necessary to ensure a smooth expansion. Such an understanding could also inform interventions to prepare dental professionals for the role. The results of this survey indicate that many dental professionals in Michigan are willing to administer the HPV vaccine if permitted by law, unlike in Arizona, Florida, and Utah, where many have opposing views [31, 36, 37]. Interestingly, we found that current students (82% of dental students and 71% of hygiene students) were more willing to administer the vaccine than practicing providers (51% of dentists and 63% of dental hygienists), suggesting that the percentage of willing providers may increase over time.

Although vaccination rates vary by state in the U.S., overall vaccination rates for HPV among adolescents and young adults continue to be low [15]. Parents have cited provider recommendations as one of the primary reasons they vaccinate their children [40]. Both medical and dental professionals, therefore, have a responsibility to educate patients about HPV prevention, especially given the rising incidence of oropharyngeal cancer associated with the virus. Dental professionals are well-positioned to deliver the HPV vaccine as they see 85% of children (ages 2–17) yearly in the U.S., often twice per year [41]. This timing is well suited for the HPV vaccine’s recommended two-dose schedule, 6–12 months apart. Even without administering the vaccine, dental professionals can take on the role of recommendation and education. Indeed, the AAPD also recommends that oral health care providers educate patients, parents, and guardians about the link between HPV and OPC, examine patients for signs of cancer, and consult the literature for the best approach to HPV infection prevention [7, 8]. It is important to note that while dental students will potentially be able to administer the vaccine once licensed, dental hygiene students and hygienists may not. The main role of the dental hygienist will be OPC prevention efforts that fall within their scope of practice, which include patient education in clinical and community settings, oral cancer screenings, and recommending the vaccine.

The CDC provides suggestions on how to make effective referrals to protect adolescent patients against HPV-associated diseases, even if not all dental professionals are administering the vaccine. An example of an effective recommendation is, “Now that your son is 11, he is due for vaccinations to help protect him from meningitis, HPV cancers, and whooping cough” [42]. Currently, the dental professional might consider providing information on where patients can get the HPV vaccine and follow up in a later visit, as well as be prepared for questions on why the patient will not be vaccinated immediately at the (dental) office, if that is the case.

The confidence of dental professionals in this task is crucial. In our survey, many respondents were not confident about their ability to discuss HPV infection with patients and their parents. Reasons for the lack of confidence could overlap with many of the barriers that respondents provided for their unwillingness to vaccinate, such as personal discomfort discussing sexual behavior, the expectation that physicians handle these discussions, religious concerns, and more. These same concerns were reported in studies that found a correlation between lack of knowledge and not willing to discuss HPV prevention with patients [37, 38, 43–45]. Therefore, training health care providers is an important step in gaining the confidence to discuss the topic and engage in cancer prevention. To this end, we found that respondents who had taken a previous class on HPV had greater confidence than those who had not. In a previous study, when dentists and hygienists received an HPV educational training, they reported increased preparedness to take an active role in OPC prevention [36, 37, 40, 46–49]. These barriers and others must be identified when developing strategies to prepare dental professionals to give strong recommendations, effectively address parental concerns, and ultimately, administer the HPV vaccine.

Many respondents felt that recommending the vaccine enhanced the health of their patients; over 50% of respondents in all four professional roles indicated that they “somewhat” or “strongly agreed.” Believing in the benefit of HPV vaccination is crucial, as we found that it increased respondents’ willingness to ultimately administer the vaccine. However, to our surprise, views of benefit seemed unaffected by previous coursework on HPV or years in clinical practice, suggesting that such beliefs may already have crystallized for most dental professionals due to education, messaging, or their or their family’s experiences with the vaccine. Future research might examine how to strengthen providers’ beliefs about the vaccine’s benefits, if possible.

Despite most professionals’ willingness to administer the vaccine, some did object. The top three reasons for unwillingness were “not having the training to administer the vaccine and monitor side effects,” “liability concerns,” and being “not willing or comfortable in discussing HPV with patients.” Practical issues such as the logistics of vaccine storage, limitations in health insurance coverage, and follow-up doses can be practical issues influencing professionals’ decisions. Other studies have examined preconceived roles and liability as influential factors for recommending and giving the HPV vaccine [31, 36, 37, 50] Some of these factors are related to providers’ assumptions or perceptions of parental hesitance [44, 45]. Notably, most concerns identified in our survey were logistical or legal, which can be remediated with training and legislation, whereas few of the respondents personally opposed the vaccine, which would require a different interventional approach. Nevertheless, even if some providers oppose the vaccine or refuse to administer it, nearly all seemed willing to refer patients to their primary care physician or other sources to receive it. This finding was consistent with previous studies indicating that dental professionals are interested in helping to increase vaccine acceptance [33, 36, 37, 45].

Interestingly, we found low awareness among the participants that dentists may administer the flu vaccine in some states. As aforementioned, Illinois and Minnesota currently allow dentists to administer the flu vaccine, and Oregon allows them to administer all vaccines, including for HPV. Respondents’ unawareness of an existing care delivery model with such a role for dental professionals may suppress political pressure for policy changes that could expand it. Despite the unawareness, though, most respondents in our survey were still willing to administer the HPV vaccine. As awareness of this model increases, we might expect professionals, parents, and patients to become even more comfortable with the delivery of vaccines in a dental setting.

Indeed, parents’ and patients’ willingness to receive the HPV vaccination from dental professionals is gradually changing towards acceptance [51, 52]. Previously cited concerns have related to insurance coverage, appropriate monitoring of side effects, and having adequately trained staff. Many parents have since recognized the convenience of vaccination at the dental office [51] and feel that dentists are qualified to counsel about HPV (66%) and its vaccination (73%), although a lower percentage feel in the same way about dental hygienists [52].

Our study has important limitations. As with any survey, there are concerns about the generalizability of our sample. The study was restricted to oral health care professionals in Michigan, so the findings may only partially represent dental professionals in the U.S. For dental hygienists, respondents were recruited from a statewide conference whose attendance might be unrepresentative within the state. The study also had an uneven number of professional categories. Meanwhile, even though a random sample of dentists was taken, and even though all dental and hygiene students in the state were given the opportunity to respond, volunteer bias may have resulted in a sample skewed toward or away from willingness to administer the HPV vaccine. Even so, the survey indicated support for administering the vaccine among hundreds of dental professionals in the state. Other limitations could include the sensitive nature of the topic, which may have resulted in response bias. For example, some respondents did not answer why they were unwilling to administer the HPV vaccine, perhaps due to stigmas or perceived professional expectations. Lastly, because this is a cross-sectional study, we cannot establish causality for the relationship between changes in attitudes or coursework experiences and willingness to administer the HPV vaccine.

### Public health implications

The growing awareness of the link between HPV and oropharyngeal cancers among dental professionals and the public calls for leadership in HPV infection prevention. The findings of this statewide study demonstrated the interest and willingness of licensed dentists and dental hygienists to administer and recommend the HPV vaccine. Additional training will be necessary to further prepare current and future clinicians to be confident to administer the vaccine, monitor side effects, and provide patient education. However, policies and legislation that empower dental professionals to administer the vaccine— alongside patient and parent education to increase comfort with the model—can potentially expand vaccination coverage and prevent HPV-related cancers.

## Supporting information

S1 Table (DOCX)
