# Supplementary material for: Oral health care professionals recommending and administering the HPV vaccine: Understanding the strengths and assessing the barriers
Source: PLoS One. 2021 Mar 4;16(3):e0248047. doi: 10.1371/journal.pone.0248047 (PMC7932114; doi:10.1371/journal.pone.0248047)
Supplement: S1 Table — (DOCX) [file pone.0248047.s001.docx]

The following questions pertain to your knowledge on the HPV vaccine and your attitude towards the HPV vaccine:

Thank you for participating in this study about oral cancer and the human papillomavirus (HPV) vaccine. Responding to this survey should take about 10 minutes. Your responses are completely anonymous.

1. Are you aware that some states allow dentists to administer flu vaccines?

a. Yes

b. No

1. As a dental professional, I think recommending HPV vaccines enhances the health of my patients.
   1. Strongly disagree
   2. Disagree
   3. Neutral
   4. Agree
   5. Strongly agree
2. Are you willing to administer the vaccine if allowed by law?
   1. Yes
   2. No
   3. Not sure
3. If you answered no or not sure to question #3, why are you not willing to administer the vaccine if allowed by law? Select all that apply.
   1. Religious reasons
   2. Liability concerns
   3. I don’t have the training to administer the vaccine and monitor side effects
   4. I am not willing or comfortable in discussing HPV with my patients or parents
   5. I am against the vaccine
   6. Other: _______________________
4. Are you willing to refer your patients to receive the vaccine in the office of a…?
   1. Primary care physician
   2. Pediatrician
   3. Pharmacy
   4. Dentist (if allowed)
   5. Patient’s school (where available)
   6. Other: _____________________
5. How likely are you to consider vaccinating your patients if a patient’s insurance provides adequate reimbursement?
6. Very Unlikely
7. Unlikely
8. Neutral
9. Likely
10. Very likely
11. How confident do you feel in discussing with patients about HPV infection?
    1. Completely uncertain
    2. Somewhat uncertain
    3. Neutral
    4. Somewhat confident
    5. Completely confident
12. Have you previously participated in a class or a continuing education course pertaining to HPV infection and/or the HPV vaccine?
13. Yes
14. No
15. How likely are you to enroll in future continuing education courses pertaining to HPV infection and/or the HPV vaccine?
16. Very unlikely
17. Unlikely
18. Neutral
19. Likely
20. Very likely

The following questions are about your background:

1. What is your gender?

Male____ Female____

1. What is your age?

I am ________ years old.

1. I am a:
2. Dental student
3. Dental hygiene student
4. Graduate student
5. Dentist
6. Dental hygienist
7. Specialist

If applicable, please indicate your dental specialty.

_________________________________________

1. If you are a student, please indicate what year:

DS1__ DS2__ DS3__ DS4__

Dental Graduate__

DH1__ DH2__ DH3__ DH4___

DH Graduate ___

Not a Student___

1. Do you intend to specialize after graduation or in the future? If so, please explain what area of study.

_____________________________________

1. What school(s) do you currently attend, or did you graduate from?

____________________________________

This marks the end of the survey if you are a student.

**For Dental Professionals Only**

1. How many years have you been in the dental profession?

0-5__ 6-10__ 11-16__ 17-20___

21-24__ 25 years or more___

1. What is the approximate percentage of adolescent patients you see in your practice per week?
   1. 0%
   2. 10%
   3. 25%
   4. 50%
   5. 75%
   6. 100%
   7. I am not a practicing clinician
